# *Mango*: unearthing patterns in large-scale biological data through interactive correlation analysis

**DOI:** 10.1186/s12864-026-13249-x

**Published:** 2026-08-06

**Authors:** Sverre Branders, Manfred G. Grabherr, Rafi Ahmad

**Affiliations:** Department of Biotechnology, University of Inland Norway, Holsetgata 22, Hamar, 2317 Norway

**Keywords:** Correlation analysis, DataVis, Multi-omics, Algorithm, Median-Ranked Label Encoding, Data integration, Biomedical data

## Abstract

**Motivation:**

Integrating different types of biological data is often challenging due to the presence of both numerical and categorical data. This complexity makes it harder to evaluate causal biological effects, especially when confounders like population structure, sampling methods, or multi-omics integration can lead to incorrect conclusions.

**Results:**

We introduce Mango, an interactive correlation browser designed for visually exploring any tabular data type, regardless of their distribution. Our results on genomic and transcriptomic datasets demonstrate that these correlations can effectively distinguish between biases and causal relationships in large-scale data.

**Availability and implementation:**

Mango uses a novel algorithm for non-parametric correlation of numeric to categorical data called Median-Ranked Label Encoding and is implemented in C++/Java and supported on Linux. Source code is available under the GNU GPLv3 license from [1].

**Supplementary Information:**

The online version contains supplementary material available at https://doi.org/10.1186/s12864-026-13249-x.

## Introduction

As high-throughput experimental techniques become more common in life sciences, data science tools like statistics and machine learning are increasingly vital for converting large datasets into meaningful scientific insights. Relying only on automated analyses can lead to incorrect assumptions about the data. A persistent challenge in bioinformatics is ensuring datasets are free of biases. These biases—including batch effects from lab procedures or poorly stratified cohorts—are systematic and can undermine the validity of the study’s results. As a result, numerous studies have been published reporting an effect or association, but the findings do not apply to cohorts beyond those studied [2–5]. Currently, there are no well-established techniques for detecting such effects in qualitative data. This emphasizes the importance of developing and validating new approaches to identify batch effects and biases, ensuring the accuracy and reproducibility of results. These effects are especially significant when, for example, examining phenotype associations in bacterial genomes, where clonal reproduction makes it difficult to distinguish causal from non-causal relationships [6]. Additionally, in multi-omics – a technology that’s currently leading to the discovery of life-saving therapeutics and diagnostics – batch effects can be especially complex. This is because multiple data types with different distributions are integrated from various platforms [2]. In this context, data visualization (DataVis) is increasingly recognized as a vital part of the data validation process [7]. Several software initiatives exist to lower the barrier of entry and promote reproducibility in this field, including BioPerl [8], Bioconductor [9], Bioconda [10], etc. These software collections still require significant domain expertise to choose and use appropriate packages for particular biological problems. Graphical applications can further lower these barriers, making the software more accessible to researchers with different scientific backgrounds. Many domain-specific graphical software tools exist, such as EpiViz [11], which implements a Shiny-based [12] web interface that is compatible with popular Bioconductor packages.

Comparing two variables, whether discrete (such as text labels), two numeric variables, or a numeric and a discrete variable, allows for a quick assessment to determine whether the correlation is desirable or not. For example, if a clinical measurement from a sample is highly correlated with the diagnosis, then this correlation is considered desirable. Conversely, if these measurements are also highly correlated with, for example, cohorts, then this suggests that the measurements are cohort-specific rather than indicative of the disease. In the case of the latter, both cohorts and measurements will exhibit high correlation. An intuitive way to assess and explore data is through visualization.

Mango is a user-friendly standalone tool designed for exploring correlations in tabular data with minimal technical knowledge. It utilizes metadata where each row represents a sample, each column a metadata variable, and each cell an observed value. Mango addresses this by: (a) creating a correlation matrix that illustrates the similarity and redundancy among variables; (b) visually presenting these results through an interactive Graphical User Interface (GUI). This GUI enables users to access data at various levels: a high-level view featuring an interactive correlation matrix with customizable row orderings, and a detailed, zoomable view for examining individual variables.

## Algorithm

Mango aims to quantify the association between metadata variables. It does this by using correlation coefficients, allowing for numerical-to-numerical, categorical-to-categorical, and categorical-to-numerical comparisons. Before applying each method, missing values are removed from the data. Numerical data is automatically detected if all the valid values are numbers.

### Numerical-to-numerical correlation

For the association of numerical variables, Mango uses the non-parametric Pearson’s correlation coefficient [13] as follows. Given two numerical variables of size * n*

$$X=\left\{\left({x}_{1},\cdots\:,{x}_{n}\right)|{x}_{i}\in\:\mathbb{\:}\mathbb{R}\right\}$$

and

$$Y=\:\left\{\left({y}_{1},\cdots\:,{y}_{n}\right)|{y}_{i}\in\:\mathbb{\:}\mathbb{R}\right\}$$

the sample correlation coefficient is computed as

$${r}_{XY}=\frac{{\sum\limits_{i=1}^{n}}({x}_{i}-{\overline{x}})({y}_{i}-{\overline{y}})}{\sqrt{{\sum\limits_{i=1}^{n}}{({x}_{i}-{\overline{y}}})^{2}}\:\sqrt{{\sum\limits_{i=1}^{n}}{({y}_{i}-{\overline{y}})}^{2}}}$$

where * x̄* and * ȳ* are the sample means. Correlation significance is tested using the Fisher transformation

$$F\left({r}_{s}\right)=\:\frac{1}{2}ln\frac{1+{r}_{s}}{1-{r}_{s}}$$

then the approximately normally distributed z-score

$$\:z=F\left({r}_{s}\right)\sqrt{\frac{n-3}{1.06}}$$

and a *p*-value can be computed using the standard normal distribution.

### Categorical-to-categorical correlation

Mango quantifies the association between non-numeric variables using Cramér’s V method [14] as follows. Given two categorical variables

$$A=\:\left\{\left({a}_{1},\cdots\:,{a}_{n}\right)|{a}_{i}\in\:\mathbb{\:}\mathbb{S},\mathbb{\:}\left|\mathbb{S}\right|=r\right\}$$

and

$$\:B=\:\left\{\left({b}_{1},\cdots\:,{b}_{n}\right)|{b}_{j}\in\:\mathbb{\:}\mathbb{T},\mathbb{\:}\left|\mathbb{T}\right|=k\right\}\:$$

of sample size * n*, the chi-squared statistic is calculated by constructing a contingency table

$$\boldsymbol{F}=\left[\begin{array}{ccc}{f}_{\mathrm{1,1}}&\cdots&{f}_{1,j}\\\vdots&\ddots&\vdots\\{f}_{i,1}&\dots&{f}_{i,j}\end{array}\right]$$

where

$${f}_{i,j}=\sum_{n}\left({A}_{n}={A}_{i},{B}_{n}={B}_{j}\right),$$

then

$${\chi\:}^{2}=\sum_{i,j}\frac{{\left({f}_{i,j}-\frac{{f}_{i}{f}_{j}}{n}\right)}^{2}}{\frac{{f}_{i}{f}_{j}}{n}}.$$

Using $\:{\chi\:}^{2},$ Cramér’s coefficient is computed as

$$V=\mathrm{m}\mathrm{a}\mathrm{x}(0,\frac{{\chi}^{2}}{n}-\frac{(k-1)(r-1)}{(n-1)}).$$

Since this coefficient tends to overestimate the strength of association, it is bias-corrected according to

$$\stackrel{\sim}{V}=\sqrt{\frac{V}{\mathrm{m}\mathrm{i}\mathrm{n}(\stackrel{\sim}{k}-1,\stackrel{\sim}{r}-1)}}$$

with

$$\stackrel{\sim}{k}=k-\frac{{\left(k-1\right)}^{2}}{n-1}$$

and

$$\stackrel{\sim}{r}=r-\frac{{\left(r-1\right)}^{2}}{n-1}$$

In addition, $\:{\chi\:}^{2}$ is used to compute a *p*-value using the cumulative chi-squared distribution with $\:\left(k-1\right)\left(r-1\right)$ degrees of freedom using the lower incomplete gamma series expansion method.

$${F}_{{\chi}^{2}}\left(x;k\right)=\frac{\gamma\left(\frac{k}{2},\frac{x}{2}\right)}{{\Gamma}\left(\frac{k}{2}\right)}$$

where the lower incomplete gamma function $\:\gamma\:$ is computed via series expansion as

$$\gamma\left(a;x\right)=\frac{1}{a}\sum\limits_{i=0}^{{\infty}}\frac{{x}_{i}}{\left(a+i\right)!}$$

iteratively until convergence. The result is adjusted to account for the exponential decay in the tail of the incomplete gamma function as

$$\gamma\left(a;x\right)={e}^{-x}+\gamma\left(a;x\right)$$

### Categorical-to-numerical correlation

Associations between a numeric and non-numeric variable are calculated by first discretizing the numeric variable using what we call “Median-Ranked Label Encoding (MRLE)”. Given a numerical variable of size * n*

$$X=\left\{\left({x}_{1},\cdots,{x}_{n}\right)|{x}_{i}\in\mathbb{\:}\mathbb{R}\right\}$$

and a categorical variable of size * n*

$$A=\left\{\left({a}_{1},\cdots,{a}_{n}\right)|{a}_{i}\in\mathbb{\:}\mathbb{S},\mathbb{\:}\left|\mathbb{S}\right|=r\right\},$$

the numerical variable * X* is first grouped by category in * A* as follows

$${\forall\:}_{s}\in\:\mathbb{S}\mathbb{\:}{\mathrm{V}}_{s}=\left\{{X}_{i}\right|{A}_{i}=\mathrm{s}\}.$$

Next, the group-wise medians are calculated as

$${m}_{s}=median\left({V}_{s}\right)$$

after which, the categories are sorted by their group medians. With a total order $\:\prec\:$ defined over $\:\mathbb{S}$:

$${s}_{1}\prec{s}_{2}\Leftrightarrow{m}_{{s}_{1}}<{m}_{{s}_{2}}$$

Finally, the original categorical labels are replaced with their corresponding median-sorted ranks. Let $\:rank\left(s\right)$ be the position of * s* in the ordered list $[{s}^{\left(1\right)},{s}^{\left(2\right)},\dots\:,{s}^{\left(k\right)}]$, then:

$${z}_{i}=rank\left({\mathrm{A}}_{\mathrm{i}}\right)$$

Then the MRLE-encoded categorical variable $\:A\:is$

$$Z=({z}_{1},{z}_{2},\dots\:,{z}_{n})\in\:{\{\mathrm{0,1},\dots\:,k-1\}}^{n}.$$

[15]. For correlations, we use the same Cramér’s V method as for categorical-to-categorical comparisons. Since MRLE depends on the number of categories in the categorized data set, we do not compute *p*-values directly to avoid circular dependencies. Instead, we perform *N* rounds of permutation testing (*N* = 5000 by default), and count *f*, the number of times the null hypothesis yields a higher chi2 value than the hypothesis. For computational efficiency, we implement an early termination scheme [16] in cases where the *p*-value can not possibly be significant after correcting for multiple testing, and assign *p* = 1 instead. The final *p*-value is *f*/*N*, which we Bonferroni-correct for multiple hypothesis testing if *f* > 0. For the distributions of correlations and *p*-values on a null hypothesis (permuted) data set based on Avershina et al., see Supplementary Figs. 1 and 2.

## Methods

### Benchmarking data sets

The Avershina et al. (AMR) dataset includes genomic and minimum inhibitory concentration (MIC) data of 58 Norwegian and 28 Indian *Escherichia coli* isolates collected between 2015 and 2019 as described in the original publication [17]. Variants were called using snippy [18] (v4.6.0) with default parameters. Subsequently, Saguaro [19] was used to generate cacti, modelling local phylogenetic homology without a priori hypotheses.

The NCBI dataset was constructed by selecting 7834 *E. coli* isolates with available MIC antibiotic susceptibility phenotypes from the NCBI isolates browser [20]. Additional metadata was collected from the BioSample database using the Entrez utility, including geolocation and detailed antibiogram data. The data was filtered to remove sparse (> 40% missing) and low information (> 90% unique) variables. After filtering, the data contained 6538 isolates and 31 variables.

### Dimensionality reduction

Numerical data were normalized, and categorical data were label-encoded using sklearn [21] (v. 1.3.2). Dimensionality reduction was performed using principal component analysis (PCA), and t-distributed stochastic neighbor embedding (t-SNE) implemented in sklearn, and uniform manifold approximation and projection [22] (UMAP).

## Results

**Fig. 1:**
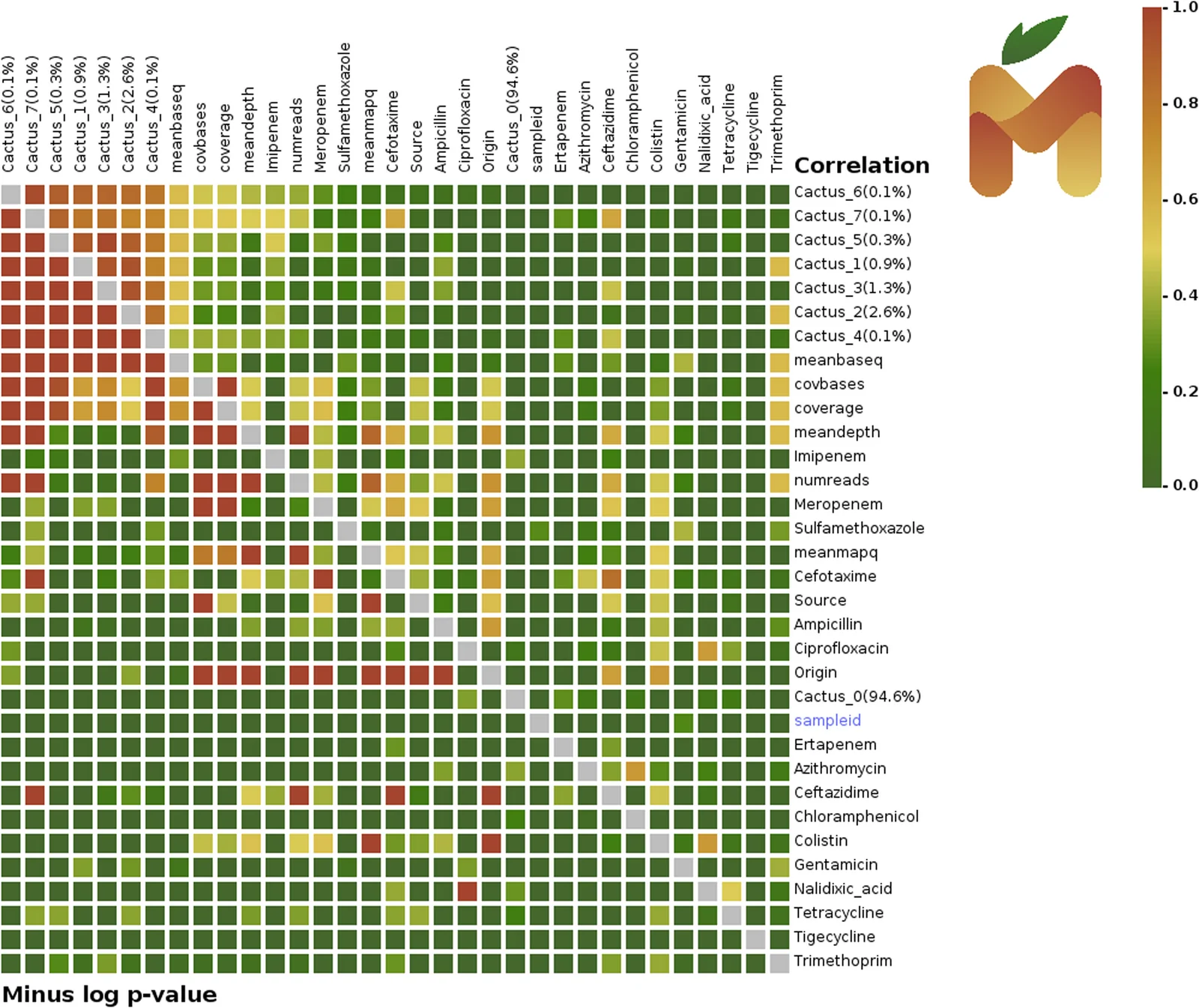
Correlation view in Mango. The matrix is sorted based on correlations to cactus 6 (top row). The top right triangle displays the correlations with color coding, while the bottom left triangle shows the *p*-values

For illustration, we used Mango on a publicly available dataset (Avershina et al. dataset) for predicting resistance to β-lactams in *Escherichia coli* based on genotype-to-phenotype analysis [17]. In addition to the metadata, we analyzed the genomes of the 86 *E. coli* samples using Saguaro [19] to identify local genomic patterns, both concordant and deviant from the overall phylogeny. Saguaro produced 8 “cacti”, i.e., vectors that describe these patterns across the samples. The Saguaro cactus 0, which models 94.6% of all variable sites and is representative of the background phylogeny, is not correlated with any other variables, including the metadata and other cacti, indicating no discernible batch effects. While cacti 1 through 7 are correlated with each other, cactus 6, modeling 0.1% of all variable sites, has the highest correlations with phenotypic susceptibility (Fig. 1).

Mango also reveals clearer correlations, such as between read coverage and the number of reads. These variables also slightly correlate with the sample origin, indicating possible differences in sequencing protocols that warrant further investigation.

**Fig. 2:**
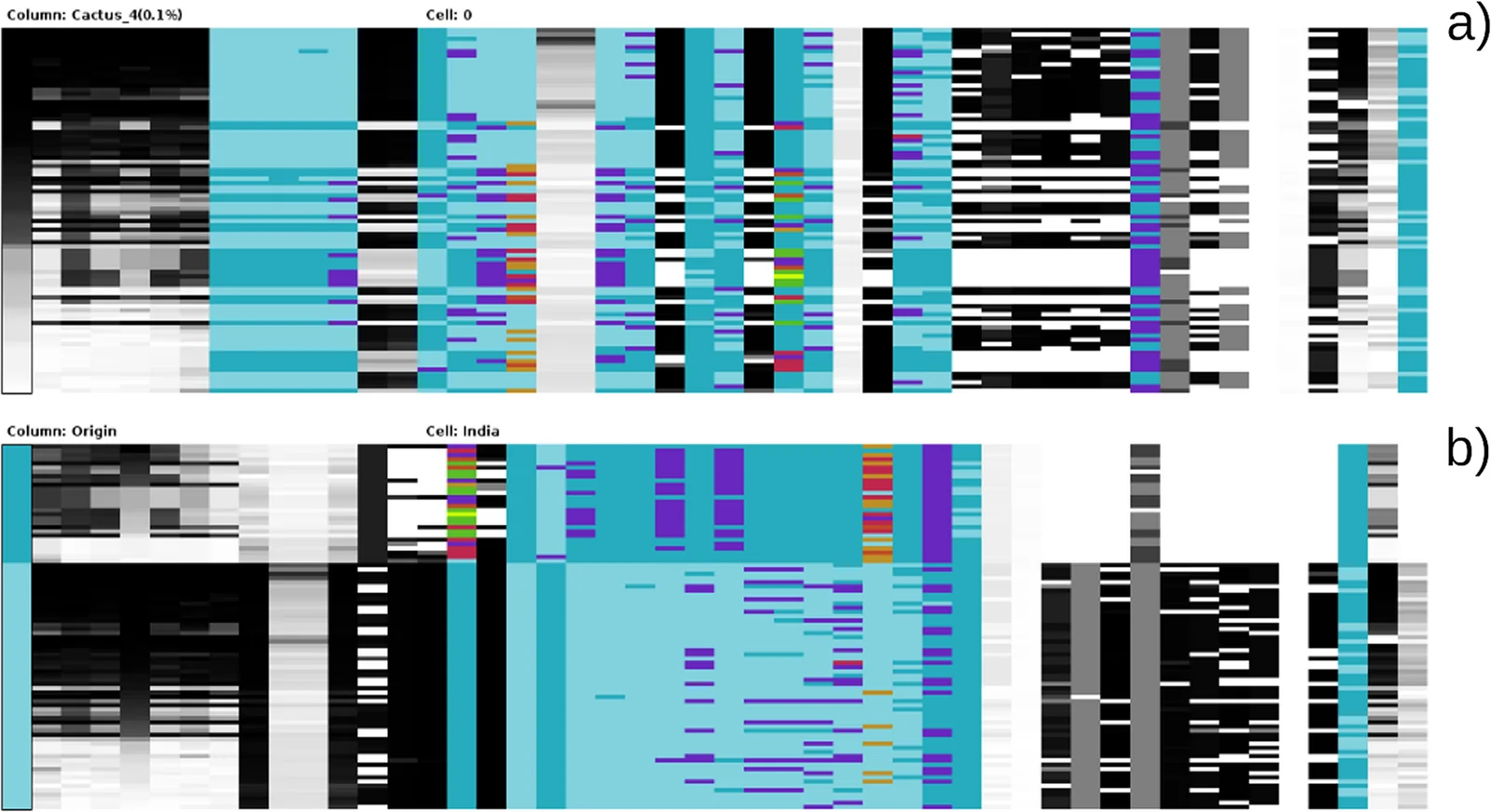
Column view in Mango. The column view is sorted by cactus 4 (**a**) and sorted by data origin (**b**). Numerical data is shown on a grey scale, e.g., columns 1 through 8, which contain values between 0 and 1. Text labels are coded into 28 colors, with colors reused as needed to accommodate more distinct labels. Hovering the mouse over a cell displays the column header and the cell’s content

Figure 2 shows the corresponding column view, sorted by cactus 4 (Fig. 2a), and by sample origin (India or Norway, Fig. 2b). This visualization presents individual elements organized into columns and rows. Numerical data is shown in a grey scale, scaled to the minimum and maximum values in each row. For text data, Mango assigns colors to each unique text category. For example, the sample source category “blood” is light blue, “urine” is purple, etc. The order of columns is based on the selected column in the correlation view, and all columns can be sorted by any column in the view, where numerical columns sorted numerically and text columns sorted alphanumerically.

**Fig. 3:**
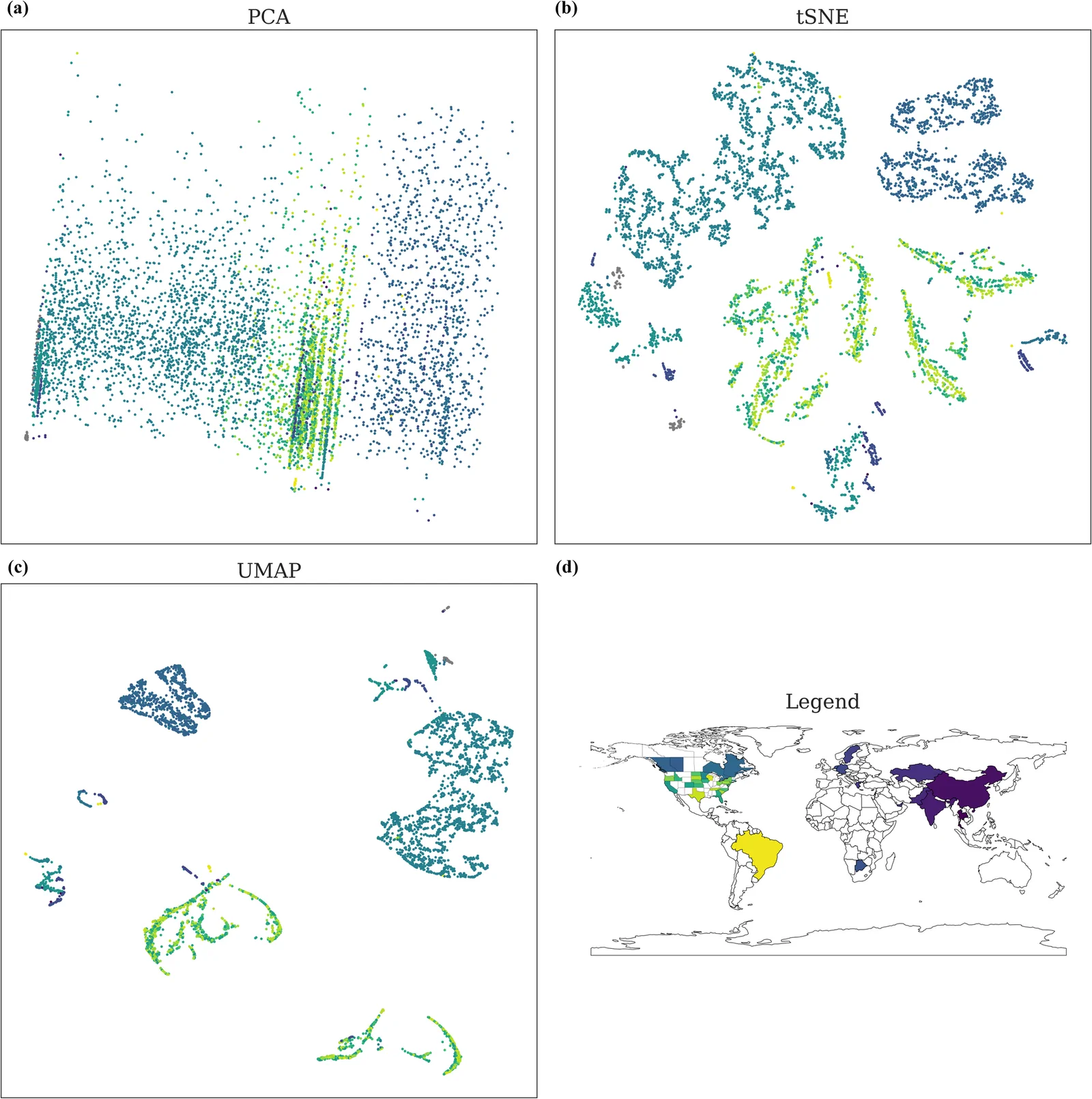
Dimensionality reduction plots of the NCBI and Avershina et al. datasets. Shown are PCA (**a**), tSNE (**b**), and UMAP (**c**). The NCBI dataset is colored by location (**d**). The distinct structure in the dimensionality reduction plots indicates that geographic location is a major factor influencing the resistance profile of *E. coli*

Mango was also run on a larger dataset (the NCBI dataset) of publicly available *E. coli* isolates collected from various geographic locations, along with their phenotypic susceptibility or resistance to antibiotics. This dataset reveals a high correlation between the origin of the isolates and their phenotypic susceptibility (e.g. erythromycin, rho = 0.78, *p* < 10^− 5^), suggesting possible batch effects. For example, the resistance profile against erythromycin shows the highest correlation with “Location” (rho = 0.79). These trends identified by Mango are also evident in the structure of the dimensionality reduction plots, which are colored by the variable with the strongest correlation (Fig. 3).

**Fig. 4:**
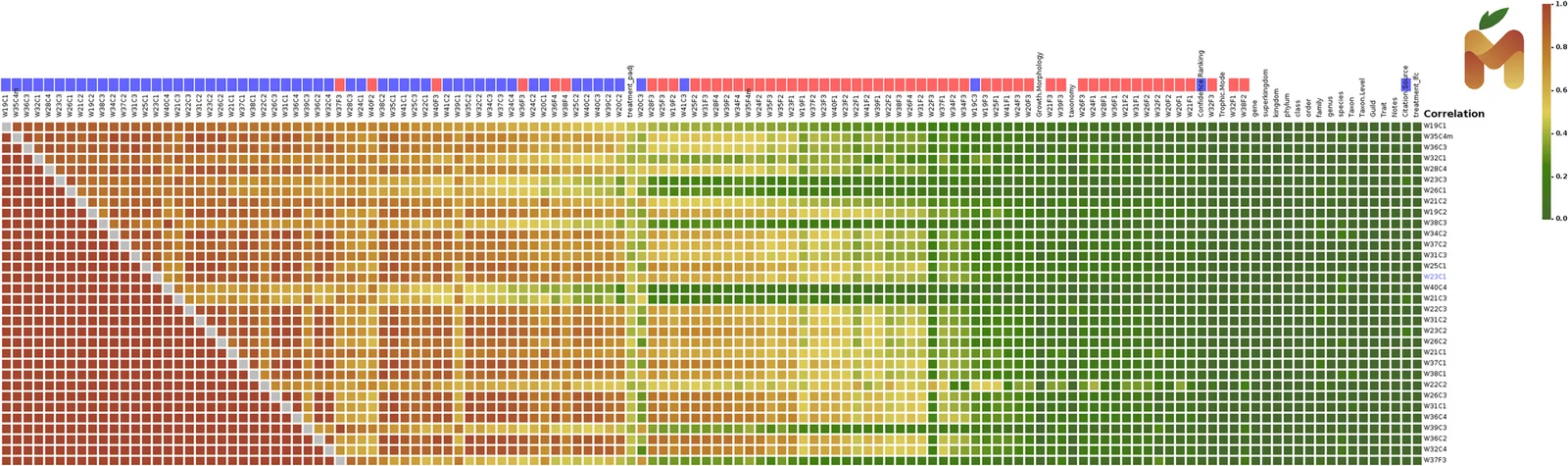
RNA-Seq expression of fungal populations in the roots of Norway spruce trees. One cohort, marked in blue, was nutrient-limited, while the other cohort, marked in red, was nutrient-enriched. The cohorts correlate with each other but are not related to any metadata (see right end of the plot)

We next applied Mango to a larger RNA-Seq data set of fungal gene expression in Norway spruce roots, where one cohort was nutrient-enriched, and the control cohort was nutrient-limited [23]. Almost instantly, Mango confirmed the key findings from the paper: the gene expression of fungi in the spruce varies significantly between the two groups but isn’t linked to any of the metadata (see right end of the plot). Figure 4 highlights the top correlations, with control samples shown in blue and fertilized samples in red.

## Discussion

Biological data sets are not only becoming larger, but also more complex and multi-modal. These data sets may aim to integrate data from different platforms, like genomics, transcriptomics, epigenetics, proteomics, spatial information, clinical data etc. In order to gain insight and discovery from these complex data, researchers increasingly rely on machine learning approaches which are able to find relationships and associations in these multi-dimensional data. The challenge, however, is ensuring that these analyses find meaningful associations rather than those caused by confounding factors [7]. Given the scale and cost required to procure biological samples and omics data, optimal study design is often too costly, increasing the risk of batch effects [24]. As demonstrated with an *E. coli* dataset (NCBI dataset), where genomic information was used to predict antibiotic resistance, the results in reality predict the location where the samples were collected. Mango’s interactive GUI makes it easy to identify such unwanted predictions with just a single mouse click, indicating that other analysis methods might be more effective for predicting antibiotic resistance from genotypes.

A popular graphical tool designed specifically for associating microbial genomic information to phenotypic traits is Scoary2 [25]. This tool enables the analysis of tens of thousands of traits, and its data visualization features can facilitate the discovery of previously unknown bacterial genotype-phenotype connections. However, this tool has two main limitations that Mango addresses: (a) it violates the assumptions of Fisher’s test, meaning that resulting correlation values can only be interpreted as arbitrary scores; and (b) its pairwise comparison algorithm can only handle binary phenotypic events, not continuous or Brownian motion-type transitions, which limits the types of data that can be integrated. Continuous traits can be binarized, but this poses a risk of improper phenotypic classification or data being discarded when it does not fit a Gaussian mean, often requiring manual intervention and expertise to overcome.

Visualizing and exploring large data sets can be challenging due to the large number of possibilities in which data can be presented. Single numbers, such as numerical correlations, can also be misleading because they do not show the structure of the underlying data. Here, we introduce Mango, an interactive visualization and analysis tool that presents data at two levels: (a) correlations between data columns based on text-to-text, text-to-numeric, and numeric-to-numeric comparisons; (b) a more detailed column view, where each data point is accessible. Being able to compute correlations between heterogeneous data entries offers an efficient way to browse data systematically, while access to the most detailed level allows for deeper exploration. Additionally, Mango can serve as a pre-screening tool to estimate the strength of signals within the data, as demonstrated by the Norway spruce fungal gene expression dataset, which can motivate more in-depth analyses in early stages of research projects.

The user interface is designed to be interactive; for example, clicking on a data label in the correlation view sorts the entire matrix. To facilitate easier navigation through the two half-matrices, correlation, and *p*-value, hovering over a data point highlights the corresponding data point in both the correlation and *p*-value sections. The sorting of columns is consistent with the column view, where data can be sorted by each column. Each view can be exported as a Portable Network Graphics (PNG) file for presentation, sharing, and analysis.

In conclusion, Mango is an interactive software tool designed for the quick analysis of mixed-type datasets. It aims to rapidly and visually identify biases in the data and encourages more detailed analyses early in research projects if initial results appear promising. To our knowledge, Mango is unique due to its interactive interface and focus on ease of use, even for non-experts, which was a key development goal. It is a standalone application that requires no libraries, containers, or virtual environments, and the entire workflow is performed through simple point-and-click operations without the need for a command line or terminal. Mango’s user-friendly interface significantly reduces barriers for researchers and scientists who want to understand their data without delving into the details of bioinformatics. Additionally, Mango greatly aids in generating new hypotheses from the data, guiding subsequent experiments or analyses. It also features a novel algorithm, Median-Ranked Label Encoding, for correlating categorical and numeric variables, regardless of their distribution, addressing an important gap in data analysis. This algorithm enables direct comparison of numeric-numeric, categorical-categorical, and categorical-numeric correlations, accelerating analysis and insight generation directly within the lab. While the correlation coefficients across different comparison types are not directly comparable, showing them side-by-side provides an overview, and the *p*-values are comparable.

Furthermore, it is important to note that Mango is not limited to biomedical datasets and can be used to visualize and analyze any data as long as it is organized in a tabular format. In summary, Mango offers valuable insights into data by revealing both expected and unexpected correlations across diverse datasets, especially useful in the early stages of large research projects.

## Supplementary Information

Supplementary Material 1.

## Data Availability

Project name: Mango Project home page: [1] Operating system: Linux Programming language: C++, Java Other requirements: openjdk > = 11.0 License: GNU GPL3 Any restrictions to use by non-academics: None other than those specified in the license. The datasets analyzed in the current study are available from the following repositories: - Avershina et al.dataset: [26, 27] - NCBI dataset: [20] - Fungal gene expression dataset: enrichment tests can be accessed on the BRA web app [28], raw RNA-seq data are available in the European nucleotide archive [29].

## Abbreviations

GUI: Graphical user interface
MIC: Minimum inhibitory concentration
PCA: Principal component analysis
t-SNE: t-distributed stochastic neighbor embedding
UMAP: Uniform manifold approximation and projection
