# Supplementary material for: Mango: unearthing patterns in large-scale biological data through interactive correlation analysis
Source: BMC Genomics. 2026 Aug 6;27:915. doi: 10.1186/s12864-026-13249-x (PMC13732827; doi:10.1186/s12864-026-13249-x)
Supplement: Supplementary file 1 [file 12864_2026_13249_MOESM1_ESM.docx]

**Supplementary Figure 1**. Null model correlation distribution for numerical-to-categorical comparisons (numtex in red), categorical-to-categorical (textex in blue), and numerical-to-numerical (numnum in black). All three correlations are centered around 0, albeit at a certain spread around 0.

**Supplementary Figure 2**. Bonferroni-corrected null model p-value distribution for numerical-to-categorical comparisons (numtex in red), categorical-to-categorical (textex in blue), and numerical-to-numerical (numnum in black). While numnum (Pearson’s rho) is spread over a wider range, numtex and textex tend to assign p-values around 1.
